# Effect of catch-up growth after food restriction on the entero-insular axis in rats

**DOI:** 10.1186/1743-7075-7-45

**Published:** 2010-05-26

**Authors:** Lu-Lu Chen, Wei-Hong Yang, Juan Zheng, Xiang Hu, Wen Kong, Hao-Hao Zhang

**Affiliations:** 1 Department of Endocrinology, Union Hospital, Tongji Medical College, Huazhong University of Science and Technology, Wuhan 430022, China

## Abstract

**Background:**

Catch-up growth after food restriction (CUGFR) is characterized by a significant change in food intake which could theoretically lead to the change in glucagon-like peptide-1 (GLP-1) secretion that consequently results in altered functions of pancreatic islets.

**Methods:**

Experimental rats were divided into two groups. Rats in CUGFR group were put on food-restriction for 4 weeks, and then allowed full access to food for 0, 2, 4 weeks respectively while rats in the control group were offered *ad libitum *access to food. Plasma glucose, insulin and GLP-1 level during OGTT were measured in all the rats. Moreover, morphology of intestinal mucosa, number of L cells, beta cell mass, incretin effect and the expression of GLP-1 receptor (GLP-1R) gene in the islets were also determined.

**Results:**

The size of pancreatic islets, insulin concentration, plasma GLP-1 concentration, incretin effect, villus height-to-crypt depth ratio and L cells were all significantly decreased in CUGFR group at the end of a 4-week food-restriction period as compared with the controls. Insulin concentration and the villus height-to-crypt depth ratio were increased and finally exceeded the level of the control group over a 4-week catch-up period. Nevertheless, at the conclusion of the study, islet size, L cells number, plasma GLP-1 concentration and incretin effect increased but failed to reach the levels of the controls.

**Conclusion:**

CUGFR decreases incretin effect and disturbs the entero-insular axis partially by decreasing GLP-1 concentration, which might be responsible for the increased risk of metabolic disorder during CUGFR.

## Background

The term 'catch-up growth' was first introduced by Prader in 1963[1], and following studies have shown that nutritional rehabilitation after malnutrition could motivate catch-up growth, which was characterized by rapid growth following a temporary period of growth retardation[2] and apparent insulin resistance[3]. A relationship between low birth weight and impaired glucose tolerance late in life has been epidemiologically identified[4] and the association between insulin resistance and impaired function of the pancreatic beta cells in adulthood has been confirmed[5]. Because of these findings, the link between catch-up growth and later metabolic disorder has become a subject of active investigation[6].

The gastrointestinal tract is a site where the body exchanges energy with the external environment. The individuals who have undergone catch-up growth after food restriction show a significant increase in appetite and food intake during nutritional rehabilitation[7]. The change in the gastrointestinal load will inevitably lead to alteration in the function of gastrointestinal tract.

The concept that intestinal hormones or 'incretins' are involved in the augmentation of insulin secretion in response to oral compared with intravenous glucose is embodied in the classical descriptions of the 'entero-insular' axis[8]. The two major hormones of the entero-insular axis are believed to be glucose-dependent insulinotropic polypeptide (GIP) and glucagon-like peptide-1 (GLP-1)[9], which exert a number of important biological effects, including inhibition of glucagons and somatostatin, maintenance of beta cell mass, delay of gastric emptying, and inhibition of feeding[10]. Currently, the control of incretin secretion represents a major goal for new therapeutic as well as nutritional strategies for treating and/or reducing the risk of hyperglycaemic syndromes, excessive body weight and thus improving overall well-being[11].

Both human and animal studies showed that individuals who have undergone catch-up growth are at a higher risk of developing the metabolic syndrome and type 2 diabetes compared to those who did not experience catch-up growth, although the exact underlying mechanisms are not fully understood. Catch-up growth after food restriction (CUGFR) is reportedly characterized by a significant change in food intake. Whether this fluctuation of food intake leads to the change in GLP-1 secretion and consequently alteration in the pancreatic islet function remains unclear. Therefore, the aim of this study was to examine the impact of CUGFR on the entero-insular axis and the possible mechanism underlying metabolic disorder.

## Methods

### General Study design

6-week-old male Sprague-Dawley rats (Center of Experimental Animals, Tongji Medical College, Huazhong University of Science and Technology, China), weighing 180-200 g and caged singly in wire bottomed cages in a temperature-controlled room (22 ± 1°C) with a 12-h light/dark cycle, were raised on a commercial pellet diet (Center of Experimental Animals, Tongji Medical College, Huazhong University of Science and Technology, China) consisting, by energy, of 22% protein, 66% carbohydrates, and 12% fat and had free access to tap water. Animals were maintained in accordance with regulations of the institute and guidelines for the care and use of laboratory animals. The experiments were conducted after 1-week adaptation to housing conditions. All protocols of animal treatment were approved by the institutional animal ethics committee.

### Animal model of catch-up growth

**Figure 1:**
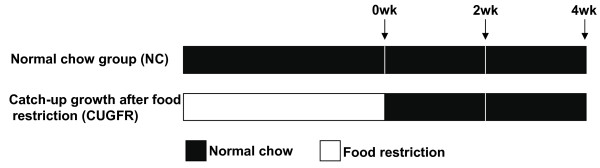
**The diagram of experiment design**. Rats were divided into NC group (n = 18) and CUGFR group (n = 18). The arrows indicate the specific days on which rats were sacrificed.

7-week-old rats (n = 36) were randomly divided into two groups (Figure 1): a normal chow group (NC group; n = 18) and a CUGFR group (n = 18). Rats in NC group were raised on a pellet diet *ad libitum *for 8 weeks and rats in CUGFR group were put on food restriction for 4 weeks at 60% of the diet intake of *ad libitum*-fed rats and then were re-fed with free access to pellet diet. Changes in food intake were determined once a day and body weight was measured every 7 days. Six rats in each group were sacrificed by decapitation 0 (the end of 4-week food restriction), 2 (catch-up growth for 2 weeks), and 4 (catch-up growth for 4 weeks) weeks after re-feeding.

### Oral glucose tolerance test

Two days before decapitation, an oral glucose tolerance test (OGTT) was performed by orally administering 0.5 g/ml glucose solution at 1 ml/100 g body weight (5 g/kg). In brief, the tip of the tail of a 20 h-fasted conscious rat was cut off and tail vein blood samples were taken, and then dextrose at 2 g/kg body weight was given by gavage. Additional tail blood samples were then drawn from the conscious rats 15, 30 and 60 min after the gavage. Blood was collected with EP tube containing EDTA (1 mg/ml) and aprotinin (5 × 10^5 ^kIU/L; Sigma, Oakville, ON, Canada) as previously reported by Reimer *et al*[12]. Diprotin A, an inhibitor of dipeptidyl peptidase IV was added to the blood sample at 34 μg/ml (Calbiochem, La Jolla, CA) to inhibit GLP-1 degradation. Blood was centrifuged at 1,600 g for 15 min at 4°C and plasma was stored at -80°C for later radioimmunoassay.

### Determination of plasma glucose, insulin and GLP-1

Plasma glucose was measured by employing a One Touch Basic blood glucose monitoring system (Lifescan Canada Ltd., Burnaby, British Columbia, Canada). Radioimmunoassay kits for insulin and active GLP-1 (7-36 amide) were obtained from Linco Research (St. Louis, MO, USA). For insulin determination, the sensitivity of the kit was < 17.22 pmol/l and intra-assay coefficient of variation was < 9.4% at 631.74 pmol/l. For GLP-1 (7-36 amide) assays, sensitivity was < 3 pmol/l and intra-assay coefficient of variation was < 12% at 40 pmol/l. Area under the curve (AUC), which reflects the area above baseline, was determined as reported by Massimino *et al*[13].

### Measurement of incretin effect

Two experiements were carried out after an overnight fast (20 hours) at an interval of 2 days. In the first experiment, rats received a 1-h OGTT (5 g/kg body weight) with plasma glucose, insulin and GLP-1 concentrations measured 0, 15, 30 and 60 min after gavage. With the second experiment, the plasma glucose profile was reproduced by a variable intravenous (IV) glucose (20% dextrose) infusion and the infusion rate was adjusted to match the glucose concentrations during the OGTT. In this experiment, venous blood was sampled 0, 15, 30 and 60 min after the start of glucose infusion for plasma glucose and insulin determination by using aforementioned method. For each rat, the incretin effect was calculated from the insulin responses during the OGTT and matched intravenous glucose infusion using the following formula[14]: 100×(Insulin AUC_OGTT_-Insulin AUC_IV glucose_)/Insulin AUC_OGTT_. This calculation eliminated the impact of glucose levels *per se*, which were matched by design.

### Immunohistochemistry and morphometry

Pancreata were resected, cleared of fat and spleen, weighed, fixed, and embedded in paraffin. 3-μm sections were deparaffinized and stained. Mouse anti-rat insulin polyclonal antibody (1:100; Boster Biotech Inc., Wuhan, China) was used for the immunofluorescence staining and tissues were processed as described[15]. Beta cell mass was morphometrically determined as described previously[16]. For negative controls, non-immune serum instead of primary antibody was used. Since we previously found that the effect of food-restriction on pancreatic islet size was greater in the head than in the tail of the pancreas (data not shown), for each animal, four representative sections of pancreas from the head region were selected for analysis.

Mucosal samples from ileum of rats were fixed in 10% buffered formalin and paraffin-embedded. Sections for histological evaluation were obtained from tissue blocks cut perpendicular to the mucosal surface, and stained with haematoxylin and eosin. Images were recorded on a digital camera (Coolpix 950, Nikon, Japan) and an Image Pro Plus software package (Media Cybernetics, Bethesda, MD, USA) was used to measure the height of 10 villi and the depth of 10 crypts in each section on randomly-selected microscopic fields [17]. The glucagon antiserum (1:100; Boster Biotech Inc., Wuhan, China) is a polyclonal antiserum which can react against enteroendocrine L cells[18]. For immunohistochemistry, tissues were processed and L cell size was calculated by morphometric analysis as previously described[19].

### RT-PCR and quantitative real-time PCR

The bile ducts of anesthetized rats were cannulated with a 27-gauge needle, and the pancreas was distended with a solution containing 2 mg/ml collagenase. Each pancreas was then incubated in a tissue culture flask at 37°C and the islets isolated according to the method reported by Gotoh *et al *[20]. Total RNA was extracted from the islets by using Simply P total RNA extraction kit (BioFlux, Hangzhou, China). Reverse transcription was performed with 1 μg of total RNA as sample material and oligo(dT)_20 _as a primer by using the first strand cDNA synthesis kit (Toyobo Co., Ltd., Osaka, Japan). The resultant cDNA was amplified using primers (synthesized by Sangon Inc. Shanghai, China), and PCR reaction was performed in a thermal cycle. Expression of the glucagons-like peptide-1 receptor (GLP-1R) gene was first detected by RT-PCR[21,22], and then real-time quantitative PCR was performed using the following profile. Primer sequences for rat GLP-1R were: 5'-GCTGGACCAGGAACTCCAACA-3' (forward) and 5'-ATGCAGATGAC CCGGATGAAG-3' (reverse). The primer sequences for beta actin gene were 5'-CGTTGACATCC GTAAAGAC-3' (forward) and 5'-TGGAAGGTGGACAGTGAG-3' (reverse). The two-step real-time quantitative PCR was done by using the SYBR Premix ExTaq (TaKaRa Biotechnology Co. Ltd., Dalian, China) and was heated at 95°C for a minimum time of 30 s, then 40 cycles at 95°C for 15 s and 60°C for 45 s in a DNA iCycler apparatus (BIO-RAD, Mississauga, ON, Canada). Results were presented as the relative expression level, which were fold ratio to the NC0 group (set to 1).

### Statistical analysis

Data analyses were performed by employing SPSS 13.0 statistical software package (SPSS, Chicago, IL, USA). Data were expressed as means ± SD. Differences between the groups were evaluated by using independent t-test, and considered as statistically significant at p < 0.05.

## Results

### Food intake and body weight

**Figure 2:**
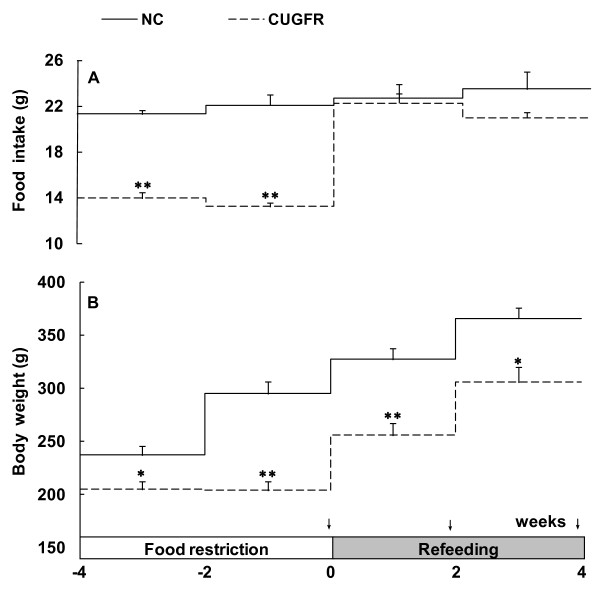
**Food intake and body weight during food restriction and re-feeding stage**. All values are expressed as means ± SD (n = 6). *P ≤ 0.05 versus NC group; **P ≤ 0.01 versus NC group. The arrows indicate the specific days on which oral glucose tolerance tests (OGTTs) were performed.

Rats in CUGFR group were fed with only 60% of the diet intake of *ad libitum-*fed rats (normal intake) during food restriction. Our previous study showed that the body weight of the rats remained roughly the same during the whole restriction period. However, decreased body weight and malnutrition resulted if they were fed with less than 60% of the normal intake (data not shown). After re-feeding, the food intake in CUGFR group increased rapidly in the first 2 weeks to reach virtually the same level as that of the control group (Figure 2-A). The body weight of these rats, however, was consistently lower than that of the control group, even four weeks after the re-feeding (Figure 2-B).

### Beta cell mass and function

**Figure 3:**
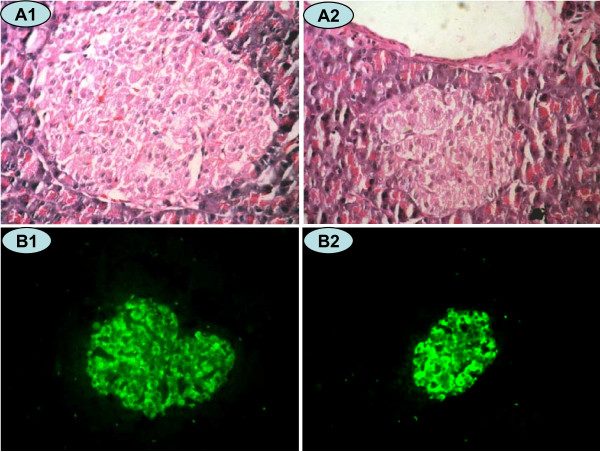
**HE Staining (×400 magnification) and insulin immunofluorescence staining (×200 magnification) in a rat pancreatic islet**. Islet cells in the NC (A1 and B1) and CUGFR (A2 and B2) groups 4 weeks after catch-up growth with HE staining (A1 and A2) and insulin fluorescence staining (B1 and B2). Green fluorescence shows cells positive for insulin.

**Figure 4:**
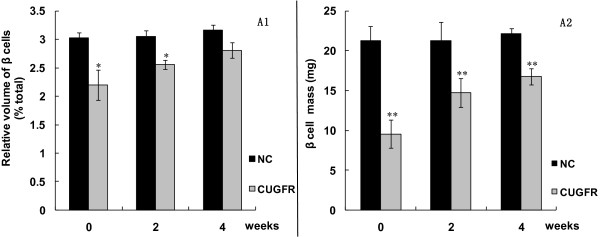
**Effect of catch-up growth on beta cell relative area and mass**. After insulin staining of rat pancreatic islet, the area positively stained for insulin was quantified by Image Pro Plus software. Values are means ± SD (n = 6). *P < 0.05 versus NC group. **P < 0.01 versus NC group.

HE and insulin fluorescence staining (Figure 3, 4) exhibited that the relative area and mass of beta cells were significantly less in CUGFR group than in NC group during food-restriction. After re-feeding, the relative area and mass of beta cells increased gradually over time, but they failed to return to level of the controls even 4 weeks after the catch-up growth.

**Figure 5:**
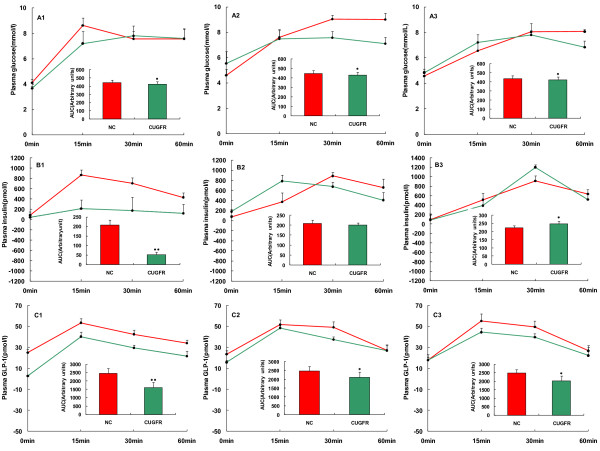
**Effect of catch-up growth on islet function and GLP-1 concentration**. Plasma glucose (A), insulin (B), GLP-1(C) levels and AUC_Insulin_, AUC_Glucose_, AUC_GLP-1 _during a 1-hour OGTT, which was conducted 0 (A1, B1 and C1), 2 (A2, B2 and C2) and 4 (A3, B3 and C3) weeks after catch-up growth in the NC (red) and CUGFR (green) groups, were measured. Data are expressed as means ± SD of six rats in each group. *P ≤ 0.05 versus NC group; **P ≤ 0.01 versus NC group.

Plasma glucose and insulin levels at the conclusion of food-restriction session were slightly lower than or similar to those of NC group 0, 15, 30 and 60 min after glucose gavage (Figure 5-A1, B1). Two weeks after the catch-up growth, plasma glucose remained lower in CUGFR group than in the NC group (Figure 5-A2) whereas plasma insulin was higher than that of controls 15 min after the gavage (Figure 5-B2). Four weeks after the catch-up, plasma glucose was still lower in CUGFR group than in NC group. However, plasma insulin levels were higher than those of controls at all time points of OGTT, especially 30 min after the gavage (Figure 5-A3, 5-B3).

### Plasma GLP-1 concentration and GLP-1R mRNA expression in islets

Food-restriction resulted in a significant decrease in plasma GLP-1 level. Following re-feeding, GLP-1 increased but did not arrive at the level of NC group even 4 weeks after the catch-up (Figure 5-C).

**Figure 6:**
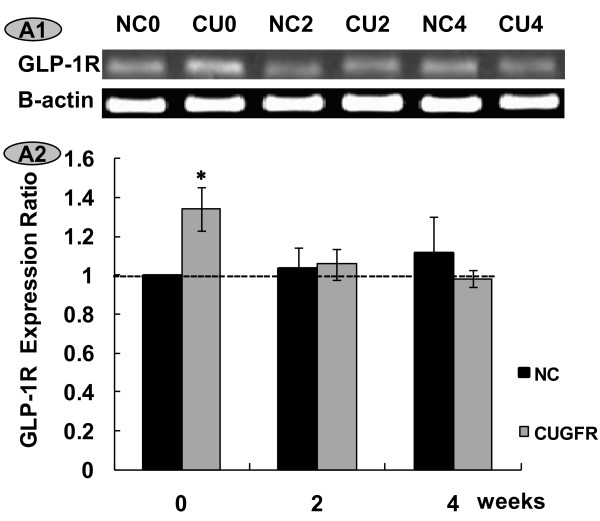
**Effect of catch-up growth on GLP-1R mRNA expression in islets**. A1. RT-PCR products from RNA extracted from rat (n = 5-6 per group) pancreatic islets. The beta actin control was comparable in all groups. A2. The statistical results of real time quantitative PCR products from RNA extracted from rat pancreatic islets. Data are expressed as means ± SD. *P ≤ 0.05 versus NC group.

As expected, electrophoresis of RT-PCR products of the pancreatic GLP-1 receptor yielded a single band (Figure 6-A1). A control PCR run with no pancreatic islet RNA or no primers yielded no product. Identity of every distinct band was confirmed by direct sequencing (data not shown). Expression levels of the GLP-1 receptor gene were compared between NC and CUGFR groups 0, 2 and 4 weeks after the catch-up growth. GLP-1R expression ratio increased significantly in the CUGFR group during food restriction. However, catch-up growth blunted this trend, and GLP-1R expression ratio declined with time and was even lower than that of NC group after 4-week catch-up growth (Figure 6-A2).

### Incretin effect

**Figure 7:**
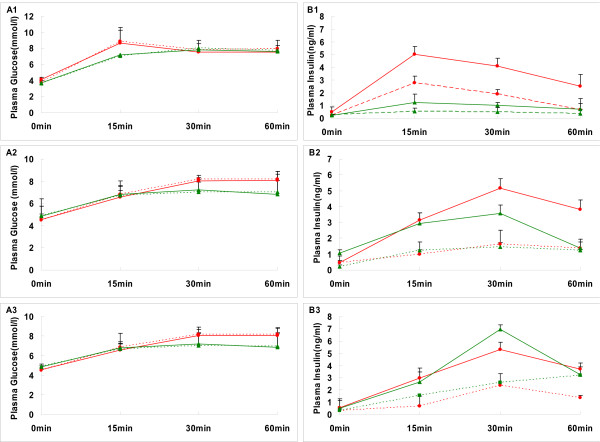
**Time course of the glucose and insulin following oral and IV glucose in the normal and catch-up growth rats**. Plasma glucose (A1, A2, A3) and insulin (B1, B2, B3) concentrations in rats of NC group (red) or CUGFR group (green) during oral glucose tolerance test (solid line) and intravenous glucose infusion test (broken lines) designed to match the oral glucose curve in six rats, were detected after catch up growth for 0 (A1, B1), 2(A2, B2) and 4 (A3, B3) weeks. Data are expressed as means ± SD.

**Figure 8:**
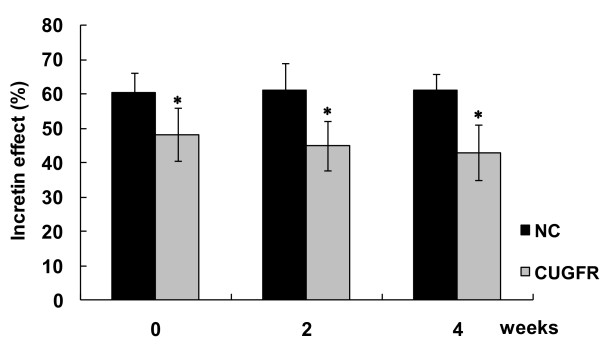
**Effect of catch-up growth on the incretin effect**. Data are expressed as means ± SD. The incretin effect was calculated by comparing the insulin response to oral and matched IV glucose load. * P ≤ 0.05 versus NC group.

The glucose concentrations were well matched between the OGTT and isoglycemic IV glucose test (IsoG IVGT) in all groups (Figure 7 A1-A3). However, the insulin response was greater after oral than intravenous glucose before or after re-feeding, which has been generally known as "incretin effect" (Figure 7 B1-B3). The incretin effect was decreased significantly after food restriction compared with NC group. After re-feeding, the incretin effect remained consistently lower in the re-feeding group till the end of the study (Figure 8).

### The morphology of intestinal mucosa and number of glucagon-positive cells in the ileum

**Figure 9:**
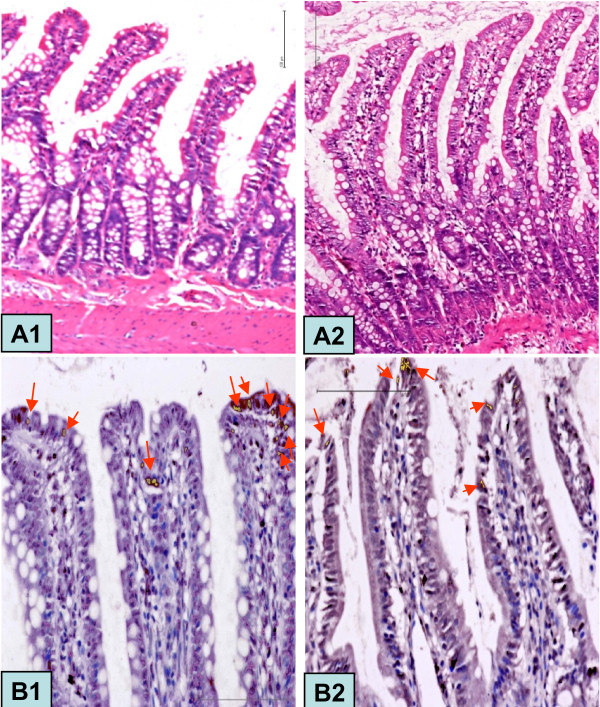
**HE Staining (×200 magnification) and glucagon staining (×400 magnification) in rat ileum**. Ileal villus observed in the CUGFR0 (A1) and CUGFR2 (A2) groups with HE staining and NC4 (B1) and CUGFR4 (B2) groups with glucagon staining. Arrow shows a glucagon-positive cell.

The morphological measurements of the rat's ileum mucosa are presented in Figure 9A and Table 1. Both villus height and villus height-to-crypt depth ratio decreased during food restriction. However, catch-up growth increased villus height and decreased the crypt depth, thereby resulting in a villus height to crypt depth ratio higher than that of NC group. Nevertheless, this trend weakened over the time of catch-up growth.

**Table 1:** Effects of catch-up growth on the morphology of the intestinal mucosa in rat ileum.

Items	Villus height (μm)	Crypt depth (μm)	Villus height:crypt depth
NC0	260 ± 26	116 ± 13	2.24 ± 0.12
NC2	268 ± 20	123 ± 21	2.18 ± 0.53
NC4	264 ± 24	128 ± 21	2.06 ± 0.41
CU0	204 ± 26^b^	111 ± 16	1.84 ± 0.63^b^
CU2	302 ± 15^a^	95 ± 22^b^	3.18 ± 0.92^b^
CU4	284 ± 25	102 ± 19^a^	2.78 ± 0.45^a^

Data are expressed as means ± SD. a P ≤ 0.05 versus NC group; b P ≤ 0.01 versus NC group.

**Figure 10:**
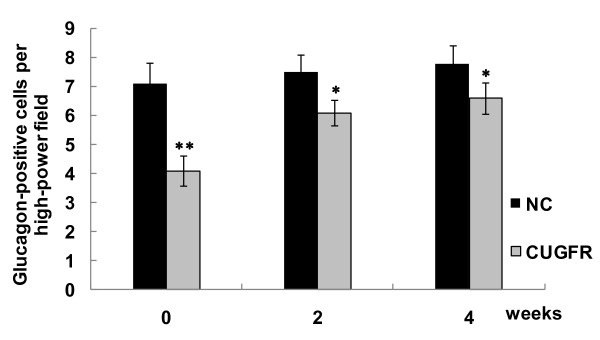
**Effect of catch-up growth on glucagon-positive cells per high-power field**. To quantify the expression of glucagon, an average of 50 sections of rat ileum from each of two animals were examined. The number of cells immunoreactive for glucagon was determined. Data are expressed as means ± SD. *P ≤ 0.05 versus NC group; **P ≤ 0.01 versus NC group.

Figure 9B and Figure 10 show that there were fewer glucagon-positive cells in CUGFR group than in NC group at the end of food restriction. Although catch-up growth partially moderated this trend, the number of glucagon-positive cells in CUGFR group failed to catch up with those of NC group even 4 weeks after the catch-up growth.

## Discussion

The importance of gut-endocrine pancreas interaction on glucose metabolism was first recognized by Claude Bernard about a century ago[23] and further conceptualized by Zunz and La Barre who introduced the term "incretins", which describe a family of hypoglycemic factors found in the extract of duodenum[24]. On the basis of understanding of the influence of the gut on pancreatic islet functions, in 1969, Unger and Eisentraut integrated these notions under a single concept of "entero-insular axis"[25]. The entero-insular axis represents a network of neural and endocrine communications between the alimentary tract and the pancreatic islets, which promotes insulin release in response to feeding. Although approximately 30% of the oral glucose-induced insulin secretory effect was accounted for by GIP, Bell and colleagues predicted the existence of GLP-1 based on their studies of the proglucagon gene[26] and subsequent investigations confirmed that GLP-1 is the most important post-prandial hormone responsible for insulin secretion[27,28].

Catch-up growth falls into two types in terms of age[2]. Early catch-up growth refers to children who are born small but catch up in weight and height in infancy or early childhood. Late catch-up growth affects children who may or may not be born small, but become stunted in infancy, childhood or adulthood and catch up later in weight, becoming "stunted-obese". The latter is more commonly seen, as it can happen in many cases, such as rapid economic development in developing countries, migration from rural to urban settings, war, famine, rehabilitation from chronic diseases, yo-yo phenomenon (weight suppression by low caloric diet, and weight rebound later), and so on[29,30]. It has been suggested that late catch-up growth may be associated with the development of type 2 diabetes and other metabolic diseases. In order to simulate the effects of late catch-up growth in human adults, the most prevalent form in developing countries, our study was conducted in mature rats and late catch-up growth was observed at different time points (0, 2, 4 weeks after re-feeding), with an attempt to examine the impact of catch-up growth on the entero-insular axis over time.

In this study, food intake and body weight of the rats as well as insulin secretion increased markedly 2 weeks after re-feeding, suggesting that catch-up growth started. Although the number of L cells and GLP-1 concentration increased at that time, the incretin effect did not parallel the increase in GLP-1, which might be ascribed to the decrease in GLP-1R. Food intake, body weight, beta cell mass and insulin concentration increased with the time of catch-up growth. On the other hand, L cell number and GLP-1 did not increase correspondingly, resulting in no increase in the incretin effect. It seems that the catch-up in the incretin effect lags far behind catch-up in food intake and body weight, which might be an important mechanism by which catch-up growth leads to metabolic disorder.

The metabolic syndrome may occur in both types of catch-up growth and most studies on the mechanisms of catch-up growth had focused on insulin resistance[31]. Although some evidence [32,33] suggests that pancreatic islet dysfunction can result from early catch-up growth, so far whether late catch-up growth also affects the beta cells has not been studied. In this study, although beta cell functions remained fundamentally normal 4 weeks after catch-up growth, in view of the lowered incretin effect and the diminished islet size, the pancreatic islets would, presumably, eventually be unable to maintain insulin secretion if the blood glucose increased excessively and rapidly over the time of catch-up growth. The question will arise as to whether in the long run this beta cell dysfunction will lead to the development of diabetes. This issue is beyond the scope of this study and will be investigated in our later studies.

This study was designed to verify whether or not the fluctuation of food intake during CUGFR leads to the change in GLP-1 secretion and thereby affects the beta cell function. Therefore, we examined the pancreatic islet size, insulin concentration and plasma GLP-1 concentration during CUGFR. Our results showed that pancreatic islet size was diminished, insulin concentration was lowered, and plasma GLP-1 concentration and incretin effect were significantly decreased at the end of the 4-week food restriction period. After 4-week catch-up growth, although both insulin and GLP-1 concentration in CUGFR group were increased, the plasma GLP-1 level still did not match that of the control group. Recent epidemiological and clinical studies demonstrated that individuals experiencing catch-up growth are more at risk of developing central obesity, glucose intolerance, type 2 diabetes mellitus and cardiovascular diseases, which are all encompassed in the definition of metabolic syndrome[30]. We believe that the changes in GLP-1 concentration and islet function play a vital role in the development of above-mentioned conditions. In accordance with our inference, Stoffers [34] was intrigued by the potential for GLP-1 agonist administration during the pre-diabetic period to ameliorate or prevent the later onset of diabetes. They used a surgical model of intrauterine growth retardation(IUGR) in which bilateral uterine artery ligation is carried out on the rat gestation day 19 and found that, at 3 months of age, all vehicle treated IUGR rats developed overt diabetes in association with a 90% decrease in beta cell mass. However, exenatide -4-treated IUGR rats maintained a normal beta cell mass and normal glucose tolerance. The normalization of blood glucose was maintained beyond 8 months of age and appeared to represent a lifelong prevention of diabetes.

Since GLP-1R, a G-protein-coupled receptor, selectively locates on the beta cells[35] to serve as a regulator of pancreatic islet mass and function, we examined the GLP-1R mRNA expression in the process of catch-up growth. We observed an increase in GLP-1R expression ratio during the food restriction period, which, together with the decrease in glucose and GLP-1 concentration, suggests that a negative feedback mechanism exists between GLP-1 and GLP-1R. This notion is consistent with the speculation put forward by Abraham *et al*[36] who speculated that the expression of the GLP-1R coupled with the expression of proglucagon and resultant GLP-1 may establish an autocrine/paracrine hormonal feedback loop that is important for some physiological functions.

GLP-1 is released from L cells of the intestines[37]. However, whether GLP-1 secretion is related to the change in the number of L-cells during CUGFR is not clear. Hence, in this study we further investigated the intestinal morphology and the change in L cell number. Some studies examined the relationship between food restriction and gastrointestinal function and demonstrated that the growth, function, and repair of gut mucosa are highly dependent on nutrient availability, and that nutritional status is associated with expression of various endogenous intestinal growth factors[38]. In line with these findings, our study showed that food restriction reduced villus height and crypt depth, resulting in fewer L cells and inevitably leading to lower plasma GLP-1 concentration and decreased incretin effect. When re-feeding starts, the increase in the gastrointestinal load motivates gut mucosal growth. The most urgent task for the body then is to create more intestinal villi so as to absorb and transport more nutrients instead of creating more L cells. Cani *et al*[19] hypothesize that L-cells, like other intestinal cells, come from common stem cells. Therefore, we can postulate that, since stem cells are available in limited amounts, the formation of L cells will be inadequate, resulting in insufficient GLP-1 concentration and weaker incretin effect even after re-feeding. However, the exact mechanisms warrant further study.

## Conclusion

Although no diabetes was observed during our study, there existed obvious secretory defects of GLP-1 and weaker incretin effect in our murine model during a 4-week catch-up growth period after food restriction, demonstrating that catch-up growth could impair entero-insular axis due to disturbed L cell formation. The detailed mechanisms remain unclear and the subsequent development of diabetes cannot be predicted at present. Therefore, further studies are needed to establish the link between the catch-up growth-related change in the entero-insular axis and diabetes mellitus.

## Competing interests

The authors declare that they have no competing interests.

## Authors' contributions

LLC and WHY were responsible for the design and overall performance of the study as well as data analysis and preparation of the manuscript. JZ, XH, WK and HHZ were all involved in the design of this study. All authors read and approved the final manuscript.
